# Online health information seeking preferences among pregnant women: a discrete choice experiment

**DOI:** 10.3389/fpubh.2026.1857953

**Published:** 2026-06-19

**Authors:** Jing Xu, Liyan Xie, Qingqing Wang, Yanxia Zhu, Yuhong Li

**Affiliations:** 1 School of Nursing, Anhui Medical University, Hefei, Anhui, China; 2 Department of Nursing, Anhui Women and Children’s Medical Center, Hefei, Anhui, China; 3 Department of Nursing, The First Affiliated Hospital of Anhui Medical University, Hefei, Anhui, China

**Keywords:** consumer health information, discrete choice experiment, internet, preferences, pregnant women

## Abstract

**Objective:**

This study aims to investigate the online health information seeking preferences of pregnant women to provide guidance for optimizing prenatal care information services.

**Methods:**

From August to September 2025, a discrete choice experiment was conducted among 342 pregnant women recruited by convenience sampling from two tertiary hospitals in Anhui Province, China. All attributes and levels were identified through literature review, qualitative interviews and expert consultations. A D-efficient design was employed to create 18 choice sets, which were randomly divided into two blocks. A mixed logit model was used to analyze choice preferences, based on which we calculated relative importance (RI), willingness to pay (WTP) and performed scenario simulation.

**Results:**

The attribute that participants valued most was the information interaction function (RI = 29.80%), followed by information content (RI = 27.01%), cost per payment (RI = 26.56%), information access method (RI = 8.73%), and information format (RI = 7.89%). Pregnant women preferred online consultation (*β* = 1.16, *p* < 0.001), knowledge about fetal growth and development (*β* = 0.59, *p* < 0.001), lower costs (*β* = −0.03, *p* < 0.001), independent search + personalized recommendation (*β* = 0.27, *p* < 0.001), and image + text + video (*β* = 0.23, *p* = 0.003). The estimated WTP for online consultation was 42.72 CNY, and this service increased the predicted choice probability by 52.40%.

**Conclusion:**

Tailoring the provision of prenatal care information services to the actual preferences of pregnant women can improve their satisfaction with online prenatal care services and their adherence to health advice.

## 1 Introduction

Pregnant women will face multiple challenges, including physiological changes, psychological stress, and shifts in social roles. Adequate information support can help alleviate the anxiety and uncertainty caused by pregnancy. Prenatal education provides pregnant women with strategies for coping with pregnancy, childbirth, and early parenting (1), covering a wide variety topics such as fetal growth, healthy lifestyles, antenatal examination, preparation for childbirth, and breastfeeding. It has been shown to play a positive role in reducing fear of childbirth, enhancing self-efficacy, lowering cesarean section rates, and decreasing the use of epidural anesthesia (2, 3). Ensuring that pregnant women have access to accurate prenatal care information is essential for improving self-care capacity and safeguarding maternal and fetal health.

The ways in which pregnant women access prenatal care information are changing. Compared to traditional sources of health information, online health information can break through the constraints of time and space, offering high accessibility, convenience, and cost-effectiveness. Research indicates that the Internet has become a significant source of health information for pregnant women. A study showed that 95% of Swedish women use the Internet to obtain health information during pregnancy (4). In China, 81.5% of women began to search for online health information from their first trimester (5). Online health information seeking can help pregnant women establish healthy lifestyles, obtain social support, and build confidence in making health decisions (6–10). However, there are still defects in the accuracy, readability and comprehensiveness of online health information (11, 12), which may cause concerns and confusion among pregnant women. Pregnant women still regard healthcare professionals as the most reliable source of health information (13) and expect to access credible online health information.

As of September 2024, China had a total of 3,340 Internet hospitals, providing more than 100 million online medical consultations annually (14). Internet hospitals integrate functions including appointment scheduling, online consultations, medical report inquiry, and health management, serving as a new platform for health education (15). By leveraging Internet hospitals to provide prenatal care information, healthcare professionals can not only ensure the professionalism of the information, but also broaden the channels for disseminating prenatal care information. However, pregnant women reported low usage rates and satisfaction scores for online services provided by public hospitals (16). Medical institutions at all levels urgently need to optimize service delivery and increase service utilization among pregnant women. Whitworth et al. (17, 18) emphasized that healthcare professionals must fully understand the information needs and preferences of pregnant women in order to provide high-quality and responsive prenatal education. Clarifying the preferences of pregnant women regarding the content, format, and methods of accessing online health information will help optimize prenatal care information services and enhance the effectiveness of health education in prenatal care.

Discrete Choice Experiment (DCE) is a stated preference measurement method widely applied in clinical treatment decisions (19), healthcare service utilization (20), and health policy research (21). By simulating real decision-making scenarios, it generates a series of choice sets composed of attributes and levels, allowing participants to make selections that reveal their underlying preferences (22). Following the utility maximization principle (23), participants tend to choose the option they perceive as having higher utility. Apart from preference quantification, DCE can also reflect how changes in relevant factors affect preferences and choice probabilities. Numerous studies have investigated the current state of online health information seeking behaviors among pregnant women (24–28), including search frequency, access channels, concerned health topics, facilitators and barriers, as well as expectations for relevant services. Nevertheless, few published studies have used quantitative preference data to identify their relative preferences and trade-offs for online health information services, especially evidence from DCEs. Therefore, this study aims to use DCE to systematically measure the online health information seeking preferences of pregnant women and to generate recommendations for optimizing the provision of online prenatal care information services.

## 2 Methods

Based on the good research practice reports of joint analysis proposed by the International Society for Pharmacoeconomics and Outcomes Research (ISPOR) (29, 30), this study utilized DCE to investigate the preferences of pregnant women for online health information. DCE is primarily achieved through the following four steps: identification of attributes and levels, experimental design, data collection, and statistical analysis.

### 2.1 Identification of attributes and levels

#### 2.1.1 Literature review

In this study, a literature review served as the initial step to identify attributes and levels. We conducted a systematic search of eight databases for relevant literature published prior to October 31, 2024, including the China National Knowledge Infrastructure, Wanfang Database, VIP Database, China Biology Medicine disc, PubMed, Web of Science, Embase, and Wiley Online Library. The literature search was conducted in both Chinese and English, using search terms such as “health information,” “online health information,” “information seeking behavior,” “information need,” and “information preference,” as well as variants of these terms combined with phrases such as “pregnant women,” “pregnancy,” “gestation,” and “gestation period.” Studies were excluded if they (1) were duplicates; (2) were unavailable in full text; (3) were reviews, theses, or conference papers; or (4) were not in Chinese or English. The initial search yielded 1,823 articles. After removing duplicates and irrelevant studies, 191 remained. Following title and abstract screening and full-text review, this study ultimately included 21 articles. Seven attributes were extracted (Supplementary Table S1).

#### 2.1.2 Qualitative interview

To enrich the set of attributes and levels, semi-structured interviews were carried out between November and December 2024 at a tertiary hospital and a community health service center in Anhui Province, China. Participants were pregnant women recruited via purposive sampling according to factors such as age, educational background, and gestational age. The researcher had long-term clinical practice in obstetrics and had built good rapport with the pregnant women. All interviews took place in quiet, private rooms. The interview outline was formulated in line with the research aims and literature review outcomes (Supplementary Table S2). Before each interview, participants were fully informed about this study, guaranteed anonymity and confidentiality, and provided informed consent. During interviews, participants were encouraged to share their thoughts freely, while nonverbal cues such as body language and emotional changes were observed and documented. Each audio-recorded interview lasted 20 to 40 min. A total of 22 pregnant women were interviewed (Supplementary Table S3). Based on the interview results, the “cost per payment” and “information interaction function” were added (Supplementary Table S4).

#### 2.1.3 Expert consultation

To determine the final attributes and levels for inclusion, two rounds of expert consultations were conducted via e-mail between April and June 2025. The expert panel consisted of 12 members from relevant fields (obstetrics, nursing, midwifery, health economics, and health informatics) (Supplementary Table S5). During this phase, experts were tasked with evaluating the appropriateness of the proposed attributes and levels, as well as the clarity of their definitions. They also reorganized and standardized duplicate or ambiguously defined attributes. Additionally, experts were permitted to add or remove attributes and levels based on their clinical and research experience. Based on expert feedback, we revised three attributes, removed two attributes, added two levels, and formulated clear definitions for all attributes and levels. Ultimately, five attributes were identified, including information content, information access method, information format, information interaction function, and cost per payment. Each attribute had 3–4 levels. For detailed information about the attributes and levels, see Table 1.

**Table 1:** Attributes and levels.

Attribute	Level
Information content*	Prenatal care knowledge
	Prenatal examination
	Fetal growth and development
	Childbirth and postpartum care
Information access method	Independent search
	Personalized recommendation
	Independent search + personalized recommendation
Information format	Audio + text
	Images + text
	Images + text + video
	Video
Information interaction function	Comment
	AI question-answering
	Community interaction
	Online Consultation
Cost per payment (CNY)	0
	10
	20
	50

*Prenatal care knowledge includes online health information related to pregnancy physiological symptoms, mental health, physical activity, diet, nutrition, weight management, pregnancy complications. Prenatal examination includes online health information related to the selection and importance of prenatal examination items, examination procedures, precautions, timing, interpretation of results, and health insurance policies. Fetal growth and development includes online health information related to fetal growth milestones, self-monitoring of fetal movements, and abnormalities of the fetus and its appendages. Childbirth and postpartum care includes online health information related to preparation before childbirth, precautions and cooperation during childbirth, breastfeeding, newborn care, postpartum care, and postpartum recovery.

### 2.2 Experimental design

Experimental design refers to the process of selecting specific methods to construct alternative scenarios and choice sets after determining attributes and levels. There were 768 alternative scenarios in this study (4^4^ × 3 = 768). If this study had used a full factorial design, it would have been impractical to ask a single participant to complete a vast number of choice sets. A systematic review concluded that 61% of empirical studies in health economics required participants to complete 9–16 choice sets (22). Fatigue is a common symptom among pregnant women. To avoid exacerbating the physiological and cognitive burden on pregnant women, this study generated a D-efficient fractional factorial design with zero priors using the dcreate routine in Stata 17.0. The design comprised 18 choice sets, which were evenly and randomly divided into two blocks. The second choice set in each block was repeated as a test set for assessing the internal validity of responses, and data from these repeated sets were excluded from the main analysis. If a participant gave inconsistent answers across the two identical choice sets, their data were excluded. Each participant was randomly assigned to one version containing 10 choice sets. During pregnancy, most women actively seek health information online rather than choosing not to obtain any information. Therefore, no opt-out option was incorporated in this study. As illustrated in Table 2, each choice set contained two alternatives. An unlabeled DCE was adopted to ensure participants made decisions based on trade-offs across all attributes, rather than focusing on labels (31).

**Table 2:** Example choice set.

Attributes	Option 1	Option 2
Information content	Prenatal care knowledge	Childbirth and postpartum care
Information access method	Personalized recommendation	Independent search
Information format	Image + text + video	Audio + text
Information interaction function	Community interaction	AI question-answering
Cost per payment	20 CNY	0 CNY
Which option do you prefer?	□	□

### 2.3 Data collection

In this study, two tertiary hospitals in Anhui Province, China, were selected for investigation. Pregnant women were recruited by convenience sampling. The inclusion criteria for participants were as follows: (1) pregnant women; (2) age ≥ 18 years; (3) possessing basic literacy skills; and (4) voluntary participation with informed consent. The exclusion criteria were those who (1) were unable to understand choice tasks; (2) planned to terminate the pregnancy; (3) were diagnosed with severe mental illness or cognitive impairment; or (4) required clinical treatment during acute exacerbation of pregnancy complications.

This study used the method proposed by Orem et al. (32) to calculate sample size (*N*), which depends on the maximum number of levels across all attributes (*c*), the number of choice sets (*t*), and the number of alternatives excluding the opt-out option (*a*). The specific formula is: *N* > 500 × *c*/(*t* × *a*). In this study, *c, t,* and *a* were 4, 9, and 2, respectively. Therefore, the minimum sample size required for each questionnaire version was 112 cases. Since we designed two versions of the questionnaire, the total sample size was required to be no less than 224 cases. Questionnaires were excluded if they failed the internal validity check (29 questionnaires were deleted), exhibited pattern-based responses (6 questionnaires were deleted), or had a missing item rate of ≥ 10%. Ultimately, data from 342 valid questionnaires were included in this study.

In order to verify the rationality and comprehensibility of the choice sets, we conducted a pilot study with 30 participants in July 2025. All participants indicated that the attributes and levels were clearly defined and the number of choice sets was appropriate. The formal survey was conducted from August to September 2025 via paper questionnaires. The questionnaire consisted of three sections: (1) study introduction; (2) demographic questions to collect sociodemographic information and pregnancy-related characteristics; and (3) the DCE choice sets (see Supplementary materials for a complete questionnaire). Informed consent was obtained from all participants before data collection.

### 2.4 Statistical analysis

This study used Microsoft Excel 2021 for data entry and formatting, and Stata17.0 for data analysis. Descriptive statistics of the participants’ characteristics were presented as frequencies and percentages. A mixed logit model with 2,000 Halton draws was constructed to analyze overall preference. The dependent variable was the participant’s choices between two alternatives (option 1 or option 2). In the model specification, cost per payment was treated as a continuous variable and specified as a fixed parameter. The remaining attributes (information content, information access method, information format, and information interaction function) were specified as random parameters and dummy-coded, with one level set as the reference. All random parameters were assumed to follow independent normal distributions. The overall model significance was assessed using the log-likelihood (LL) statistic. The Akaike information criterion (AIC) and Bayesian information criterion (BIC) were used to test model fit.

The relative importance (RI) was calculated to clarify the contribution of each attribute to the overall utility, where $A_{k}$ represented the difference in utility between the highest and the lowest level of the *k*-th attribute. The equation was as follows:

$$RI_{k}=(\frac{A_{k}}{\sum_{k=1}^{5}A_{k}})\times 100\%$$

Willingness to pay (WTP) for each non-price attribute was defined as the negative ratio of the attribute coefficient to the price coefficient. This study adopted the nlcom command to derive WTP values. The Delta method was applied to assess the uncertainty of WTP estimates. The equation was:

$$\mathrm{WT}P_{\text{non}-\text{price attribute}}=-\frac{\beta_{\text{non}-\text{price attribute}}}{\beta_{\text{price attribute}}}$$

Additionally, we conducted scenario simulation analysis to calculate predicted uptake rates, which revealed the probability that pregnant women would select given services. The significance level of all data analysis was set at *p* < 0.05.

## 3 Results

### 3.1 Characteristics of pregnant women

As shown in Table 3, the final analysis included 342 participants, with an average age of 30.47 ± 3.98 years. Most pregnant women were under 35 years old (81.9%) and lived in urban areas (86.8%). Regarding educational level, 47.4% held a bachelor’s degree. Over half (69.9%) were still employed. 46.8% reported a monthly per capita household income of 5,001–10,000 CNY. In terms of pregnancy-related data, the gestational age distribution was relatively balanced, with 28.4%, 34.2%, and 37.4% in the first, second, and third trimesters, respectively. More than two-thirds (68.1%) were nulliparous. Women without adverse pregnancy history (76.9%) and those without pregnancy complications (77.8%) accounted for the majority.

**Table 3:** Characteristics of participants.

Characteristic	*n*	Percentage (%)
Age		
<35 years old	280	81.9
≥35 years old	62	18.1
Educational level		
Junior high school	16	4.7
Senior high school	15	4.4
Technical secondary school	14	4.1
Junior college	102	29.8
Bachelor’s degree	162	47.4
Master’s degree and above	33	9.6
Employment status		
Employed	239	69.9
Non-employed	103	30.1
Living area		
Urban	297	86.8
Rural	45	13.2
Monthly per capita household income (CNY)		
≤5,000	69	20.2
5,000–10,000	160	46.8
>10,000	113	33.0
Gestational age		
First trimester	97	28.4
Second trimester	117	34.2
Third trimester	128	37.4
Parity		
Nulliparous	233	68.1
≥1	109	31.9
Planned to be pregnant		
No	95	27.8
Yes	247	72.2
History of assisted reproduction		
None	323	94.4
Yes	19	5.6
Adverse pregnancy history		
None	263	76.9
Yes	79	23.1
Pregnancy complications		
None	266	77.8
Yes	76	22.2

### 3.2 Overall preference information

Table 4 shows the results of the mixed logit model and RI. The final model statistics were: LL = −1,843.303, AIC = 3,732.605, BIC = 3,887.284. All attributes had a statistically significant impact on pregnant women’s preferences for online health information seeking (*p* < 0.05). Information interaction function was the most influential attribute (RI = 29.80%). The RI of the other attributes were: information content (RI = 27.01%), cost per payment (RI = 26.56%), information access method (RI = 8.73%), and information format (RI = 7.89%). Regarding information interaction function, pregnant women most preferred online consultation (*β* = 1.16, 95% CI: 0.95 to 1.38). Compared with prenatal care knowledge, pregnant women had a strong preference for knowledge about fetal growth and development (*β* = 0.59, 95% CI: 0.42 to 0.77). The negative coefficient of cost per payment indicated that pregnant women preferred online health information with lower costs (*β* = −0.03, 95% CI: −0.03 to −0.02). Pregnant women also preferred for “independent search + personalized recommendation” (*β* = 0.27, 95% CI: 0.12 to 0.41) and “image + text + video” (*β* = 0.23, 95% CI: 0.08 to 0.39).

**Table 4:** Results of the mixed logit model and RI.

Attributes and levels	Coefficient (β)	SE	*p*	95% CI		RI (%)
Cost per payment (CNY)	−0.03	0.002	<0.001***	−0.03	−0.02	26.56
Information content (Reference Level: Prenatal care knowledge)						27.01
Prenatal examination	0.37	0.084	<0.001***	0.20	0.53	
Fetal growth and development	0.59	0.089	<0.001***	0.42	0.77	
Childbirth and postpartum care	0.43	0.083	<0.001***	0.27	0.59	
Information access method (Reference Level: Personalized recommendation)						8.73
Independent search	0.18	0.066	0.006**	0.05	0.31	
Independent search + personalized recommendation	0.27	0.072	<0.001***	0.12	0.41	
Information format (Reference Level: Image + text)						7.89
Audio + text	0.02	0.081	0.815	−0.14	0.18	
Image + text + video	0.23	0.079	0.003**	0.08	0.39	
Video	0.15	0.095	0.109	−0.03	0.34	
Information interaction function (Reference: Comment)						29.80
AI question-answering	−0.03	0.082	0.756	−0.19	0.14	
Community interaction	0.34	0.086	<0.001***	0.17	0.51	
Online consultation	1.16	0.110	<0.001***	0.95	1.38	

*p < 0.05, **p < 0.01, ***p < 0.001, Sample size = 342, Observations = 6,156.

### 3.3 WTP

WTP of pregnant women for each attribute is presented in Table 5. Pregnant women had a strong preference for the information interaction function: they were willing to pay 12.45 CNY for community interaction and 42.72 CNY for online consultation. Regarding information content, pregnant women were willing to pay 21.71 CNY for knowledge about fetal growth and development. If the information access method changed from “personalized recommendation” to “independent search + personalized recommendation”, WTP would be 9.77 CNY higher. Additionally, regarding information format, compared with “image + text”, they were willing to pay 8.56 CNY for “image + text + video”.

**Table 5:** Results of WTP.

Attributes and levels	WTP (CNY)	*p*	95% CI	
Information content (Reference Level: Prenatal care knowledge)				
Prenatal examination	13.41	<0.001***	7.34	19.48
Fetal growth and development	21.71	<0.001***	14.95	28.48
Childbirth and postpartum care	15.71	<0.001***	9.75	21.66
Information access method (Reference Level: Personalized recommendation)				
Independent search	6.66	0.006**	1.90	11.43
Independent search + personalized recommendation	9.77	<0.001***	4.36	15.19
Information format (Reference Level: Image + text)				
Audio + text	0.70	0.815	−5.16	6.56
Image + text + video	8.56	0.003**	2.92	14.21
Video	5.59	0.109	−1.33	12.51
Information interaction function (Reference: Comment)				
AI question-answering	−0.93	0.756	−6.81	4.95
Community interaction	12.45	<0.001***	6.08	18.81
Online consultation	42.72	<0.001***	34.74	50.69

*p < 0.05, **p < 0.01, ***p < 0.001.

### 3.4 Scenario simulation analysis

According to the results, the information interaction function, information content, and cost per payment are the primary factors influencing pregnant women’s choice of online health information services. We used comments, prenatal care knowledge, 50 CNY, personalized recommendation, and “image + text” as baseline levels. Holding other baseline levels constant, the probability of pregnant women accepting online health information services offering online consultations increased by 52.40%. When the information content was adjusted to focus on fetal growth and development, the probability of pregnant women accepting this option increased by 28.74%. When the cost per payment was reduced from 50 CNY to 20 CNY, 10 CNY, and 0 CNY, the probability of selection increased by 38.73, 49.66, and 59.22%, respectively. Table 6 presents the results of the scenario simulation analysis.

**Table 6:** Results of scenario simulation analysis.

Scenario	Information content	Information access method	Information format	Information interaction function	Cost per payment (CNY)	Selection probability change
Baseline	Prenatal care knowledge	Personalized recommendation	Image + text	Comment	50	—
Scenario 1	Prenatal examination	Personalized recommendation	Image + text	Comment	50	+18,07%
Scenario 2	Fetal growth and development	Personalized recommendation	Image + text	Comment	50	+28.74%
Scenario 3	Childbirth and postpartum care	Personalized recommendation	Image + text	Comment	50	+21.07%
Scenario 4	Prenatal care knowledge	Independent search	Image + text	Comment	50	+9.05%
Scenario 5	Prenatal care knowledge	Independent search + personalized recommendation	Image + text	Comment	50	+13.23%
Scenario 6	Prenatal care knowledge	Personalized recommendation	Image + text + video	Comment	50	+11.61%
Scenario 7	Prenatal care knowledge	Personalized recommendation	Image + text	Community interaction	50	+16.79%
Scenario 8	Prenatal care knowledge	Personalized recommendation	Image + text	Online consultation	50	+52.40%
Scenario 9	Prenatal care knowledge	Personalized recommendation	Image + text	Comment	0	+59.22%
Scenario 10	Prenatal care knowledge	Personalized recommendation	Image + text	Comment	10	+49.66%
Scenario 11	Prenatal care knowledge	Personalized recommendation	Image + text	Comment	20	+38.73%

## 4 Discussion

Online consultation was the most preferred information interaction function, which provided an efficient way for pregnant women to obtain prenatal care information. As gestational age increases, pregnant women may experience symptoms such as fatigue, edema, and lower back pain (33, 34). Frequent hospital visits increase their physical burden. By initiating online consultations via Internet hospitals, pregnant women can receive professional assessments and recommendations directly, thereby reducing the number of unnecessary offline visits and associated costs. While using pregnancy-related apps and social media, pregnant women may feel anxious if they cannot access relevant information or are exposed to negative content (35). Online consultation for information verification helps them get personalized guidance to avoid falling into cognitive confusion. Qin et al. (36) found that the working atmosphere and performance bonuses were the primary factors influencing the delivery of health education services by healthcare professionals. Therefore, institutions should foster a positive work environment for health education, establish reasonable incentive mechanisms with bonuses and honors, encourage healthcare professionals to participate in online consultations, and provide specialized training to enhance their e-health literacy and online communication skills (37). Pregnant women were willing to pay 42.72 CNY for online consultation, and the availability of this service raised the predicted choice probability by 52.40%. For resource-constrained public hospitals, prioritizing the addition of moderately priced online consultation functions may better improve pregnant women’s satisfaction.

In this study, pregnant women also showed a preference for community interaction. Jiang et al. (38) found that pregnant women who joined peer groups typically exchanged, verified, and sought advice regarding pregnancy, which made them perceive higher social support. However, it is difficult to verify whether this health information is reliable. This suggests that medical institutions should establish official online communities for pregnant women within internet hospitals, invite healthcare professionals to participate in management, and regularly post vetted prenatal care information within the groups. Institutions can also explore the use of intelligent assistance tools to automatically identify risk terms and misinformation, prompting healthcare professionals to provide timely explanations and corrections.

Fetal growth and development emerged as the topic of greatest concern for pregnant women. Larsson et al. (39) and Zhu et al. (40) reported similar conclusions. This result can be explained first from the perspective of maternal-fetal attachment, which refers to the emotional bond established between pregnant women and the developing fetus from early pregnancy (41, 42). The fetus relies on the mother to provide the living environment, while the mother invests strong emotions and attention in the fetus. Second, growth and development indicators serve as key reflections of fetal health and act as early warning signs for adverse pregnancy outcomes, including fetal growth restriction, macrosomia, and low birth weight. Acquiring knowledge about fetal growth and development facilitates early intervention. Additionally, according to our findings, health education on other topics also should not be overlooked. Prenatal care is a systematic health management process, requiring healthcare professionals to provide pregnant women with continuous and comprehensive health information support (43). Therefore, Internet hospitals shall take knowledge of fetal growth and development as the core of online health information services, integrate internal resources and develop a systematic knowledge matrix that includes prenatal care, prenatal examinations, delivery and postpartum care. As content focusing on fetal growth and development increased the acceptance probability by 28.74%, placing popular science modules about fetal development in prominent positions can improve the utilization rate of online health information services among pregnant women.

The results showed that pregnant women were more likely to obtain free or cheaper online health information. Wang et al. (44) similarly found that women favored AI chatbots offering free health information. Pregnancy imposes additional financial burdens, including expenses related to prenatal examinations, nutritional supplements, and infant supplies. In contrast, pregnant women tend to view paid health information as a non-essential expense. Social media is a popular medium for disseminating health information. It aggregates a wealth of free health information and has become a key channel for pregnant women to access and communicate health information (40). To improve the quality of free health information available to pregnant women, public hospitals can establish official accounts on social media platforms and synchronize health education updates across their Internet hospitals and social media channels. Obstetric professionals should strengthen communication with pregnant women (45, 46) and employ health information quality assessment tools (47) to evaluate the quality of free online health information, thereby recommending trustworthy sources to pregnant women. For pregnant women with a certain level of financial means and health pursuits, medical institutions can try to provide paid services to meet their needs, which offer personalized health management, customized health education prescriptions, and regular follow-up.

Among pregnant women, “independent search + personalized recommendation” was the most popular method to access information. Xu et al. (27) similarly found that women with gestational diabetes expect to build an online platform with a simple search function and personalized recommendations. A systematic review reported that the use of digital health technologies directly influenced women’s empowerment (48), whether as skills acquisition systems or health education portals. Empowerment of pregnant women is positively correlated with their health literacy (49). When pregnant women search for online health information, it helps foster a sense of empowerment, which in turn enables them to better access, understand, and apply health information. Furthermore, in response to immediate needs, independent searching is a quick way for pregnant women to obtain information and relieve concerns (50). Based on data such as demographic characteristics, gestational age, and health status, personalized recommendations can proactively provide health information to address the diverse and staged health information needs (26, 51). Therefore, Internet hospitals should adopt user-centered designs, such as quick search, classification navigation, and personalized recommendation functions, to help pregnant women access targeted information efficiently. However, the RI of information access method was 8.73%, which was substantially lower than information interaction function, information content and cost per payment. In the short term, Internet hospitals ought to give priority to optimizing higher-ranking attributes. Low-cost solutions, including a search function and basic recommendation algorithms, are sufficient for upgrading information access channels.

Regarding information format, despite being the least influential attribute, pregnant women preferred the level of “image + text + video”, and its WTP was 8.56 CNY. According to the dual coding theory, humans process information through two systems: one processes visual information, and the other processes verbal information (52). When information is presented in a combination of text and images, both systems are activated, which enhances retention of the relevant information (53). Meanwhile, information in video format can effectively maintain the learner’s attention, which is beneficial for reinforcing memory and understanding (53, 54). This finding suggests that using varied, engaging mixed formats for online health information is a straightforward strategy to enhance online health services targeting pregnant women. Implementing a visual hierarchy in educational materials and using color contrast to highlight key content will also further improve learning outcomes for pregnant women (55).

## 5 Limitation

This study contributes to understanding pregnant women’s preferences for online health information services, but it has several limitations. First, the study employed convenience sampling, and the survey was conducted at only two tertiary hospitals in a single region. The sample consisted primarily of highly educated, urban-dwelling, nulliparous women, resulting in selection bias. These sample characteristics limit the representativeness of the study, which not only restricts the generalizability of the findings but also hinders the analysis of preference heterogeneity. Future research could involve large-scale, multi-center studies with broader coverage to explore preference heterogeneity among pregnant women from different demographic groups and regions. Second, DCE is a type of stated preference research method that simulates real-world scenarios; as such, it is subject to certain hypothetical bias. Additionally, this study did not include an opt-out option, which may have overestimated preference weights. Third, as this study examined only five key attributes, it may not fully reflect the decision-making scenarios in the real world, and the influence of other attributes on preferences may be ignored. To understand the online health information seeking preferences of pregnant women more comprehensively, future research should continue to explore relevant attributes in depth.

## 6 Conclusion

In this study, DCE was used to measure the online health information seeking preferences of pregnant women. The results indicate that information interaction function, information content, and cost per payment are the three key factors influencing the selection of pregnant women. When using Internet hospitals to provide prenatal care information services, medical institutions should encourage healthcare professionals to actively participate in online consultations and develop comprehensive health education databases for pregnant women. Additionally, they can leverage social media to enhance the professionalism of free online prenatal care information.

## Data Availability

The raw data supporting the conclusions of this article will be made available by the authors, without undue reservation.
