# Supplementary material for: Online health information seeking preferences among pregnant women: a discrete choice experiment
Source: Front Public Health. 2026 Jun 19;14:1857953. doi: 10.3389/fpubh.2026.1857953 (PMC13328091; doi:10.3389/fpubh.2026.1857953)
Supplement: Supplementary file 1 [file Table_1.docx]

Supplementary Material

Table S1 Attributes Extracted Based on Literature Review

| **Number** | **Attribute** |
| --- | --- |
| 1 | Information content |
| 2 | Information access method |
| 3 | Information format |
| 4 | Information provider |
| 5 | Community interaction |
| 6 | Interpretation of medical insurance policies |
| 7 | Mental health support |

Table S2 Interview Outline

| **Number** | **Question** |
| --- | --- |
| 1 | Since becoming pregnant, for what purposes do you access online health information? |
| 2 | Through which channels do you obtain online health information? What experiences and feelings do you have during the process of obtaining online health information? |
| 3 | Which topics of online health information do you pay attention to? What format of online health information do you prefer? |
| 4 | In your opinion, what impacts has the acquisition of online health information had on you? |
| 5 | Do you have any other requirements for the current online health information services during pregnancy? |

Table S3 Basic Information of Interviewees

| **Number** | **Age**  **(years)** | **Educational Level** | **Gestational Weeks** | **Fertility Circumstance** | **Pregnancy Complication** |
| --- | --- | --- | --- | --- | --- |
| N1 | 29 | Junior college | 32 | G2P0 | No |
| N2 | 33 | Senior high school | 32 | G1P0 | Yes |
| N3 | 29 | Bachelor degree | 34 | G1P0 | Yes |
| N4 | 34 | Master degree | 38 | G2P0 | No |
| N5 | 30 | Bachelor degree | 23 | G2P0 | No |
| N6 | 29 | Bachelor degree | 13 | G1P0 | Yes |
| N7 | 30 | Bachelor degree | 9 | G1P0 | No |
| N8 | 33 | Bachelor degree | 36 | G3P0 | Yes |
| N9 | 32 | Bachelor degree | 28 | G3P1 | No |
| N10 | 35 | Bachelor degree | 10 | G3P1 | No |
| N11 | 28 | Junior college | 12 | G1P0 | No |
| N12 | 24 | Technical secondary school | 17 | G1P0 | No |
| N13 | 31 | Bachelor degree | 25 | G1P0 | No |
| N14 | 29 | Bachelor degree | 21 | G1P0 | No |
| N15 | 26 | Bachelor degree | 26 | G1P0 | No |
| N16 | 27 | Bachelor degree | 28 | G1P0 | Yes |
| N17 | 28 | Junior college | 31 | G1P0 | Yes |
| N18 | 29 | Bachelor degree | 19 | G1P0 | Yes |
| N19 | 37 | Bachelor degree | 20 | G2P1 | No |
| N20 | 26 | Bachelor degree | 32 | G1P0 | Yes |
| N21 | 30 | Bachelor degree | 35 | G2P0 | No |
| N22 | 28 | Junior college | 25 | G1P0 | Yes |

Table S4 Attributes and Levels Revised Following Qualitative Interviews

| Number | Attribute | Levels |
| --- | --- | --- |
| 1 | Information content | Pregnancy care related  Prenatal examination related  Fetal related  Delivery and postpartum related |
| 2 | Information access method | Search engines  Medical/health information portals  Maternal apps  Social media platforms |
| 3 | Information format | Video  Text  Images  Combination of images and text |
| 4 | Information provider | Medical professionals  Dedicated maternal bloggers  Other pregnant users |
| 5 | Information interaction function | Comment  Community interaction  Online consultation |
| 6 | Cost per payment | 10 CNY  20 CNY  50 CNY |
| 7 | Interpretation of medical insurance policies | Needed  Not needed |
| 8 | Mental health support | Needed  Not needed |

Table S5 Basic Information of Experts

|  | **Age**  **（years）** | **Educational**  **Level** | **seniority** | **Research**  **Direction** |
| --- | --- | --- | --- | --- |
| A | 37 | Doctoral degree | 14 | Perinatal medicine |
| B | 39 | Master degree | 13 | Perinatal medicine |
| C | 59 | Master degree | 40 | Midwifery |
| D | 32 | Master degree | 6 | Midwifery |
| E | 50 | Master degree | 31 | Maternal-child nursing |
| F | 51 | Master degree | 29 | Maternal-child nursing |
| G | 37 | Master degree | 15 | Maternal-child nursing |
| H | 45 | Doctoral degree | 22 | Health education and health promotion |
| I | 44 | Doctoral degree | 12 | Health education and health promotion |
| J | 48 | Doctoral degree | 25 | Maternal-child nursing |
| K | 32 | Doctoral degree | 6 | Maternal-child nursing |
| L | 43 | Doctoral degree | 19 | Health economics |

**Questionnaire on Online Health Information Seeking Preferences Among Pregnant Women**

**I. Informed Consent Form**

Dear Participants:

Online resources have become one of the most important channels for pregnant women to obtain health information. This study intends to investigate pregnant women’s preferences and demands for online health information services, with the aim of providing references for the improvement of prenatal care information services. We greatly appreciate your support. There are no standard answers to the questions. Please complete this questionnaire carefully based on your real experience and genuine thoughts. This research has obtained ethical approval from the Biomedical Ethics Committee of Anhui Medical University and complies with the principles of voluntariness, confidentiality and beneficence. We guarantee that all your information will be kept strictly confidential. The collected data will only be used for research purposes and will not exert any impact on your medical care or expenses.

If you agree to participate in the study, please sign below. Thank you for your valuable participation!

Name：

Date：

**II. General Information Questionnaire**

(1) Demographic Information

1. Age: years old

2. Ethnicity: ① Han ② Other:

3. Educational Level:

① Primary School

② Junior High School

③ Senior High School

④ Technical secondary school

⑤ Junior College

⑥ Bachelor’s Degree

⑦ Master’s Degree and above

4. Occupation:

① Leaders of Party organs, state organs, mass organizations, social organizations, enterprises, and public institutions

② Professional and technical personnel

③ Administrative staff and related personnel

④ Social production and public service workers

⑤ Agricultural, forestry, animal husbandry, and fishery production and support staff

⑥ Manufacturing and related personnel

⑦ Military personnel

⑧ Unemployed

⑨ Other:

5. Employment Status: ① Full-time ② Part-time ③ On leave

6. Living Area: ① Urban ② Rural

7. Health Insurance Type:

① Basic Medical Insurance for Urban and Rural Residents

② Basic Medical Insurance for Urban Employees

③ Fully Self-funded

④ Other:

8. Your household’s average monthly income per capita is:

① <3,000 yuan ② 3,000–5,000 yuan ③ 5,001–10,000 yuan ④ >10,000 yuan

(2) Pregnancy-related Information

9. Gestational Age: weeks

10. You have given birth to children; of these, were vaginal deliveries and were cesarean sections.

11. Was this pregnancy planned? ① Planned ② Unplanned

12. Number of fetuses in this pregnancy: ① 1 ② 2 ③ 3 or more

13. History of assisted reproduction: ① None ② Yes

14. Adverse obstetric history (such as miscarriage, fetal birth defects, and stillbirth):

① None ② Yes

15. Pregnancy complications (such as anemia during pregnancy, gestational diabetes, gestational hypertension, hypothyroidism, vaginitis, and placenta previa):

① None ② Yes

**III. Choice Tasks**

To understand your preferences regarding online health information, we will present you with ten decision scenarios, each featuring two completely different options, each with its own advantages and disadvantages. Please weigh the pros and cons and select the option you prefer in each scenario. The following scenarios are hypothetical; your choice will not affect your medical care or costs. Table 1 shows you the meaning of each attribute and level. As shown in Table 2, if you prefer option 2, tick it.

Table 1 Attributes and Levels

| **Attribute** | **Level** |
| --- | --- |
| Information content* | Prenatal care knowledge |
|  | Prenatal examination |
|  | Fetal growth and development |
|  | Childbirth and postpartum care |
| Information access method | Independent search |
|  | Personalized recommendation |
|  | Independent search + personalized recommendation |
| Information format | Audio + text |
|  | Images + text |
|  | Images + text + video |
|  | Video |
| Information interaction function | Comment |
|  | AI question-answering |
|  | Community interaction |
|  | Online Consultation |
| Cost per payment (CNY) | 0 |
|  | 10 |
|  | 20 |
|  | 50 |

*Prenatal care knowledge includes online health information related to pregnancy physiological symptoms, mental health, physical activity, diet, nutrition, weight management, pregnancy complications. Prenatal examination includes online health information related to the selection and importance of prenatal examination items, examination procedures, precautions, timing, interpretation of results, and health insurance policies. Fetal growth and development includes online health information related to fetal growth milestones, self-monitoring of fetal movements, and abnormalities of the fetus and its appendages. Childbirth and postpartum care includes online health information related to preparation before childbirth, precautions and cooperation during childbirth, breastfeeding, newborn care, postpartum care, and postpartum recovery.

Table 2 Task Example

|  | Option 1 | Option 2 |
| --- | --- | --- |
| Information content | Childbirth and postpartum care | Prenatal care knowledge |
| Information access method | Personalized recommendation | Personalized recommendation |
| Information format | Image + text + video | Video |
| Information interaction function | AI question-answering | Comment |
| Cost per payment | 0 CNY | 10 CNY |
| Which option do you prefer? | □ | ☑ |

Now, please start choosing:

Choice Sets Version 1

|  | Option 1 | Option 2 |
| --- | --- | --- |
| Information content | Prenatal care knowledge | Childbirth and postpartum care |
| Information access method | Personalized recommendation | Independent search |
| Information format | Image + text + video | Video |
| Information interaction function | Community interaction | AI question-answering |
| Cost per payment | 20 CNY | 0 CNY |
| Which option do you prefer? | □ | □ |

|  | **Option 1** | **Option 2** |
| --- | --- | --- |
| Information content | Childbirth and postpartum care | Fetal growth and development |
| Information access method | Independent search + personalized recommendation | Personalized recommendation |
| Information format | Image + text | Audio + text |
| Information interaction function | Comment | Online consultation |
| Cost per payment | 50 CNY | 0 CNY |
| Which option do you prefer? | □ | □ |

|  | **Option 1** | **Option 2** |
| --- | --- | --- |
| Information content | Fetal growth and development | Childbirth and postpartum care |
| Information access method | Independent search | Independent search + personalized recommendation |
| Information format | Audio + text | Image + text + video |
| Information interaction function | Comment | AI question-answering |
| Cost per payment | 50 CNY | 0 CNY |
| Which option do you prefer? | □ | □ |

|  | **Option 1** | **Option 2** |
| --- | --- | --- |
| Information content | Prenatal examination | Prenatal care knowledge |
| Information access method | Independent search + personalized recommendation | Personalized recommendation |
| Information format | Image + text | Image + text + video |
| Information interaction function | Community interaction | Online consultation |
| Cost per payment | 20 CNY | 50 CNY |
| Which option do you prefer? | □ | □ |

|  | **Option 1** | **Option 2** |
| --- | --- | --- |
| Information content | Fetal growth and development | Prenatal examination |
| Information access method | Independent search | Personalized recommendation |
| Information format | Image + text + video | Image + text |
| Information interaction function | Community interaction | Online consultation |
| Cost per payment | 10 CNY | 20 CNY |
| Which option do you prefer? | □ | □ |

|  | **Option 1** | **Option 2** |
| --- | --- | --- |
| Information content | Childbirth and postpartum care | Fetal growth and development |
| Information access method | Independent search + personalized recommendation | Personalized recommendation |
| Information format | Audio + text | Image + text |
| Information interaction function | Online consultation | AI question-answering |
| Cost per payment | 50 CNY | 10 CNY |
| Which option do you prefer? | □ | □ |

|  | **Option 1** | **Option 2** |
| --- | --- | --- |
| Information content | Prenatal examination | Prenatal care knowledge |
| Information access method | Personalized recommendation | Independent search |
| Information format | Image + text | Video |
| Information interaction function | AI question-answering | Community interaction |
| Cost per payment | 50 CNY | 20 CNY |
| Which option do you prefer? | □ | □ |

|  | **Option 1** | **Option 2** |
| --- | --- | --- |
| Information content | Childbirth and postpartum care | Fetal growth and development |
| Information access method | Independent search | Independent search + personalized recommendation |
| Information format | Audio+ text | Video |
| Information interaction function | Comment | Community interaction |
| Cost per payment | 10 CNY | 0 CNY |
| Which option do you prefer? | □ | □ |

|  | **Option 1** | **Option 2** |
| --- | --- | --- |
| Information content | Prenatal examination | Childbirth and postpartum care |
| Information access method | Independent search + personalized recommendation | Personalized recommendation |
| Information format | Video | Images + text + video |
| Information interaction function | Online consultation | Comment |
| Cost per payment | 10 CNY | 20 CNY |
| Which option do you prefer? | □ | □ |

|  | **Option 1** | **Option 2** |
| --- | --- | --- |
| Information content | Childbirth and postpartum care | Fetal growth and development |
| Information access method | Independent search + personalized recommendation | Personalized recommendation |
| Information format | Images + text | Audio + text |
| Information interaction function | Comment | Online consultation |
| Cost per payment | 50 CNY | 0 CNY |
| Which option do you prefer? | □ | □ |

Choice Sets Version 2

|  | **Option 1** | **Option 2** |
| --- | --- | --- |
| Information content | Childbirth and postpartum care | Prenatal care knowledge |
| Information access method | Independent search | Independent search + personalized recommendation |
| Information format | Images + text | Audio + text |
| Information interaction function | Online consultation | AI question-answering |
| Cost per payment | 10 CNY | 50 CNY |
| Which option do you prefer? | □ | □ |

|  | **Option 1** | **Option 2** |
| --- | --- | --- |
| Information content | Childbirth and postpartum care | Prenatal examination |
| Information access method | Personalized recommendation | Independent search |
| Information format | Video | Images + text + video |
| Information interaction function | AI question-answering | Comment |
| Cost per payment | 10 CNY | 0 CNY |
| Which option do you prefer? | □ | □ |

|  | **Option 1** | **Option 2** |
| --- | --- | --- |
| Information content | Fetal growth and development | Prenatal care knowledge |
| Information access method | Personalized recommendation | Independent search |
| Information format | Images + text + video | Video |
| Information interaction function | AI question-answering | Comment |
| Cost per payment | 20 CNY | 10 CNY |
| Which option do you prefer? | □ | □ |

|  | **Option 1** | **Option 2** |
| --- | --- | --- |
| Information content | Prenatal care knowledge | Prenatal examination |
| Information access method | Independent search | Personalized recommendation |
| Information format | Images + text | Images + text + video |
| Information interaction function | Online consultation | Comment |
| Cost per payment | 20 CNY | 10 CNY |
| Which option do you prefer? | □ | □ |

|  | **Option 1** | **Option 2** |
| --- | --- | --- |
| Information content | Prenatal examination | Fetal growth and development |
| Information access method | Independent search | Independent search + personalized recommendation |
| Information format | Video | Images + text |
| Information interaction function | AI question-answering | Comment |
| Cost per payment | 20 CNY | 0 CNY |
| Which option do you prefer? | □ | □ |

|  | **Option 1** | **Option 2** |
| --- | --- | --- |
| Information content | Fetal growth and development | Childbirth and postpartum care |
| Information access method | Independent search + personalized recommendation | Personalized recommendation |
| Information format | Images + text + video | Audio + text |
| Information interaction function | Online consultation | Community interaction |
| Cost per payment | 50 CNY | 0 CNY |
| Which option do you prefer? | □ | □ |

|  | **Option 1** | **Option 2** |
| --- | --- | --- |
| Information content | Childbirth and postpartum care | Prenatal care knowledge |
| Information access method | Personalized recommendation | Independent search + personalized recommendation |
| Information format | Video | Audio + text |
| Information interaction function | Community interaction | AI question-answering |
| Cost per payment | 50 CNY | 10 CNY |
| Which option do you prefer? | □ | □ |

|  | **Option 1** | **Option 2** |
| --- | --- | --- |
| Information content | Childbirth and postpartum care | Prenatal care knowledge |
| Information access method | Independent search + personalized recommendation | Independent search |
| Information format | Audio + text | Video |
| Information interaction function | Community interaction | Comment |
| Cost per payment | 10 CNY | 0 CNY |
| Which option do you prefer? | □ | □ |

|  | **Option 1** | **Option 2** |
| --- | --- | --- |
| Information content | Prenatal care knowledge | Fetal growth and development |
| Information access method | Independent search | Independent search + personalized recommendation |
| Information format | Images + text | Video |
| Information interaction function | Community interaction | Comment |
| Cost per payment | 50 CNY | 20 CNY |
| Which option do you prefer? | □ | □ |

|  | **Option 1** | **Option 2** |
| --- | --- | --- |
| Information content | Childbirth and postpartum care | Prenatal examination |
| Information access method | Personalized recommendation | Independent search |
| Information format | Video | Images + text + video |
| Information interaction function | AI question-answering | Comment |
| Cost per payment | 10 CNY | 0 CNY |
| Which option do you prefer? | □ | □ |

This concludes the survey. Thank you again for your cooperation and participation! We wish you a joyful pregnancy.
